# Enterohemorrhagic *Escherichia coli* Hybrid Pathotype O80:H2 as a New Therapeutic Challenge

**DOI:** 10.3201/eid2209.160304

**Published:** 2016-09

**Authors:** Nurcan Soysal, Patricia Mariani-Kurkdjian, Yasmine Smail, Sandrine Liguori, Malika Gouali, Estelle Loukiadis, Patrick Fach, Mathias Bruyand, Jorge Blanco, Philippe Bidet, Stéphane Bonacorsi

**Affiliations:** Centre Hospitalo-Universitaire Robert-Debré (AP-HP), Paris, France (N. Soysal, P. Mariani-Kurkdjian, Y. Smail, S. Liguori, P. Bidet, S. Bonacorsi);; Institut National de la Santé et de la Recherche Médicale, Paris (N. Soysal, P. Mariani-Kurkdjian, P. Bidet, S. Bonacorsi);; Université Paris Diderot, Paris (N. Soysal, P. Mariani-Kurkdjian, P. Bidet, S. Bonacorsi);; Institut Pasteur, Paris (M. Gouali);; Laboratoire National de Réference pour les *Escherichia coli*, Marcy l’Etoile, France (E. Loukiadis);; ANSES, Maison-Alfort, France (P. Fach);; Institut de Veille Sanitaire, Saint Maurice, France (M. Bruyand);; Universidade de Santiago de Compostela, Lugo, Spain (J. Blanco)

**Keywords:** emergence, enterohemorrhagic Escherichia coli, E. coli, O80:42, Shiga toxin, hemolytic uremic syndrome, HUS, extraintestinal virulence factors, pS88 plasmid, bacteria, bacteremia, antibiotic resistance, antibiotic treatment, antimicrobial resistance, antimicrobial, enteric infections

## Abstract

We describe the epidemiology, clinical features, and molecular characterization of enterohemorrhagic* Escherichia coli (*EHEC) infections caused by the singular hybrid pathotype O80:H2, and we examine the influence of antibiotics on Shiga toxin production. In France, during 2005–2014, a total of 54 patients were infected with EHEC O80:H2; 91% had hemolytic uremic syndrome. Two patients had invasive infections, and 2 died. All strains carried *stx2* (variants *stx2a, 2c,* or *2d*); the rare intimin gene (*eae*-ξ); and at least 4 genes characteristic of pS88, a plasmid associated with extraintestinal virulence. Similar strains were found in Spain. All isolates belonged to the same clonal group. At subinhibitory concentrations, azithromycin decreased Shiga toxin production significantly, ciprofloxacin increased it substantially, and ceftriaxone had no major effect. Antibiotic combinations that included azithromycin also were tested. EHEC O80:H2, which can induce hemolytic uremic syndrome complicated by bacteremia, is emerging in France. However, azithromycin might effectively combat these infections.

## Abstract 2

This emerging clonal group harbors the extraintestinal virulence–associated plasmid pS88 and can induce invasive infections and death.

Enterohemorrhagic *Escherichia coli* (EHEC) are a subset of Shiga toxin–producing *E. coli* (STEC) that cause diarrhea and hemorrhagic colitis; illness can progress to hemolytic uremic syndrome (HUS) in 5%–10% of cases (*1*,*2*). HUS is the most frequent etiology of pediatric acute renal failure, and its lethality is 3%–5% worldwide (*1*,*3*) and 1% in France (*4*). Long-term renal injuries occur in 20%–30% of HUS patients (*1*,*3*,*5*).

EHEC serotype O157:H7 accounts for ≈60% of HUS cases worldwide (*6*,*7*). Other well-known serogroups associated with HUS include O26, O111, O145, O55, O103, O121, and O91. EHEC O80:H2 strains are rarely reported in the literature but have been detected in France. In 2013, an EHEC O80:H2 strain was responsible for a severe case of HUS with relapse associated with bacteremia (*8*). We identified several genetic traits in this isolate, such as a rare variant of the intimin gene (*eae*-ξ) and genetic determinants related to the pS88 plasmid associated with extraintestinal-virulence pathogenic *E. coli* (ExPEC) (*9*). This plasmid, mainly found in avian pathogenic *E. coli *and* E. coli* strains that cause neonatal meningitis, may partly explain the bacteremia observed, which has been reported in patients with HUS.

The occurrence of bacteremia during EHEC infections warrants antibiotic treatment for those infections. However, antibiotics usually are not recommended for EHEC infection because of the risk for worsening HUS, notably by induction of synthesis or secretion of Shiga toxin (Stx) (*1*,*10*–*12*). Therefore, bacteremia during EHEC infection represents a new therapeutic challenge. However, a 2011 outbreak in Germany linked to EHEC O104:H4 (*13*) underscored the potential benefit of certain antibiotics when HUS occurs (*14*,*15*). Thus, the use of antibiotics during EHEC infections remains a source of debate (*2*,*16*).

Our study aimed to determine the incidence rate of HUS cases associated with the singular EHEC O80:H2, describe their clinical features, and examine the molecular characteristics of the strains. In addition, we assessed the effects of different antibiotics on Stx production in representative strains.

## Materials and Methods

### Clinical Data

For this study, we considered all *E. coli* O80:H2 isolates received during January 2005–October 2014 by the Centre National de Référence Associé *Escherichia coli* (Paris, France). We then collected demographic and clinical data from patients’ medical records (e.g., age, sex, location); presence of diarrhea (with or without blood); possible source of infection; presence of neurologic or other complications (including pancreatitis, hepatitis, myocarditis, and bacteremia); whether the patient had HUS; and outcome at time of follow-up (e.g., relapse, residual renal injuries [including proteinuria and renal failure], arterial hypertension, or death). HUS was defined as anemia (hemoglobin <10 g/dL), thrombopenia (platelets <150,000/mm^3^), and renal failure (creatinine above reference for age, weight, and sex, or >0.2 protein/creatinine ratio).

### Bacteria Strains

We recovered isolates 35344 and 35431 from stool and blood cultures, respectively, of a HUS patient who was the subject of a recent case report (*8*). These strains belonged to sequence type 301 and harbored 4 intestinal virulence genes (*stx2c*, *stx2d*, *hlyA*, and *eae*-ξ) and most of the extraintestinal virulence genes carried by plasmid pS88 (*8*). The Laboratoire National de Réference pour les *Escherichia coli* (Marcy l’Etoile, France) and reference laboratories from Spain, Italy, and Germany were associated with this study and provided us with their EHEC O80 strains when available. The reference strain EDL933 (O157:H7, *stx*1a, *stx*2a, and *eae*-γ) served as the control in the study of Stx production (*17*). We stored all EHEC strains at −80°C in 5% glycerol.

### Serotyping

The Centre National de Référence des *Escherichia coli*, *Shigella*, et *Salmonella *at the Institute Pasteur (Paris, France) initially determined the O80 serogroup by using a method based on the analysis of the O antigen genes cluster *rfb* restriction fragments length polymorphism (*18*); this result was recently confirmed by O80-specific PCR with primers targeting O80 polymerase gene *wzy* (GenBank accession no. AB812032). This new PCR was included in our previously described O-serogroup multiplex PCR (*19*). We assessed specificity of the new multiplex PCR on template DNA extracted from 130 O reference strains as previously described (*20*). Primers used for the EHEC O-serogrouping multiplex PCR are provided (Technical Appendix Tables 1, 2). We determined the H serogroup by using PCR targeting the *fliC* genes (*21*).

### Molecular Characterization

Among EHEC O80 strains, we screened several genetic determinants by multiplex PCR as previously described (*22*), including intestinal virulence genes (*stx1*,* stx2*,* eae*, and *hlyA*) and extraintestinal virulence genes associated with plasmid pS88 (*sitA*,* eitB*,* cia, iss, iucC*,* iroN*,* hlyF*,* etsC*,* cvaA*, and *ompT_p_*) (*9*,*23*). We also determined the variants of stx2 (*stx2a*, stx2*b, stx2c,* and stx2*d*) and the variant of *eae* by using PCR-based methods (*24*,*25*).

To investigate the genetic diversity of the O80:H2 strains studied, we used the DiversiLab genotyping method (bioMérieux, Marcy-l’Étoile, France), which is based on PCR amplification of repeat sequences of DNA (rep-PCR) as previously described (*26*). We then compared these genotypes with representative strains of serogroups (O157, O104, O121, and O111) previously typed and recorded in our DiversiLab database (S. Bonacorsi, unpub. data).

### Effect of Various Antibiotics on Stx Production

We prepared inocula to assess Stx production in the presence or absence of antibiotics as previously described (*27*). We obtained log-phase growth of strains in brain–heart infusion broth by using overnight incubation at 37°C and then diluted the result with Luria-Bertani broth for an inoculum of 10^6^ CFU/mL. We added 3 antimicrobial agents (azithromycin, ciprofloxacin, and ceftriaxone) at final MICs of 0.5 and 0.25 for a single assay. We also tested combinations of antibiotics at a MIC of 0.5. For each strain, we also performed an antibiotic-free assay. We collected the bacterial cultures after 18 h of incubation at 37°C and centrifuged at 4°C at 2,000 rpm for 10 min. We filtered supernatants through a 0.22-µm pore-size filter (Millipore, Bedford, MA, USA) and stored at −20°C until needed.

We quantified Stx1 and Stx2 by using a chemiluminescent immunoassay for EHEC toxins (Liaison, DiaSorin, Spain). We expressed the results in relative light units, and converted each result to a concentration of Stx (ng/mL) by using a standard curve obtained by serial dilution of a highly positive sample and the manufacturer-provided positive control (70 ng/mL Stx concentration). We performed each measure 3 times.

### Statistical Analysis

We calculated means, medians, and SDs in Excel (Microsoft Corp., Redmond, WA, USA). Student paired *t*-test was used to compare means of Stx concentrations; p values <0.05 were considered statistically significant. Quantitative variables are presented as median and range or quartile range.

## Results

During January 2005–October 2014, the Centre National de Référence Associé *Escherichia coli* collected 57 strains of EHEC O80:H2 in France. These strains were isolated, mostly from stool specimens, from 54 patients; 2 and 3 isolates each were recovered from 2 patients. Clinical data were available for all but 1 patient.

**Figure 1:**
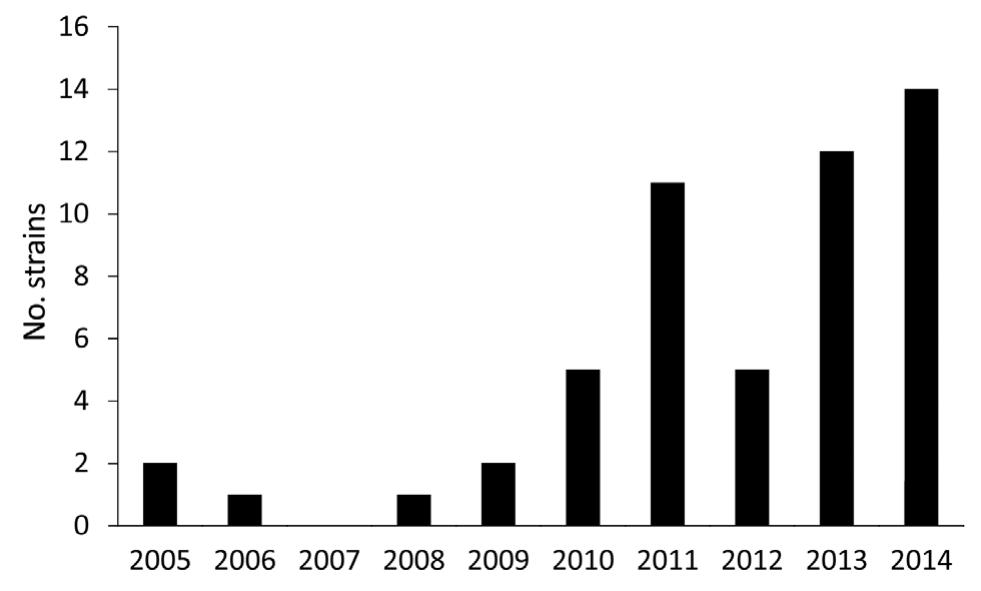
Number of enterohemorrhagic *Escherichia coli* O80:H2 strains detected annually, France, January 2005–October 2014.

**Figure 2:**
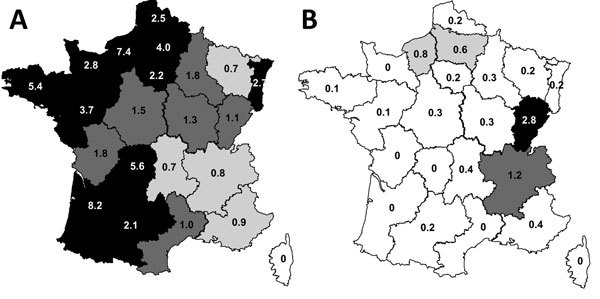
Regional 10-year cumulative incidence rates of hemolytic uremic syndrome cases caused by enterohemorrhagic *Escherichia coli* serotypes O157:H7 and O80:H2, France, January 2005–October 2014. A) Serotype O157:H7. B) Serotype O80:H2. White, <0.5 cases/100,000 children; light gray shading, 0.5–0.7 cases/100,000 children; medium gray shading, 0.8–0.9 cases/100,000 children; dark gray shading, 1–2 cases/100,000 children; black, >2 cases/100,000 children.

The spatiotemporal distribution of the O80:H2 infections clearly indicates an increased number of infections during the past 5 years (Figure 1). In 2014, the EHEC O80 serogroup was the second-leading cause of pediatric HUS in France (*4*). Most of the cases occurred during summer and the beginning of autumn. Geographic distribution of O80:H2 EHEC infections in France revealed high 10-year cumulative incidences (>1/100,000) in Franche-Comté (2.83/100,000 children) and in Rhône-Alpes (1.19/100,000 children), contrasting with the distribution of O157 infections, which are rarely detected in these areas (Figure 2).

Among the 53 patients for whom clinical data were available, 48 (91%) had HUS; 27 (51%) were male. Median age for these 48 patients was 1.2 years (range 0.2–39 years, interquartile range [IQR] 0.7–1.6 years). Only 1 adult HUS patient (a 39-year-old) was reported. The 5 (9%) non-HUS patients were largely older (1, 2, 6, 21, and 40 years old). Among HUS patients, fever was present in 45%; median leukocyte count was 13,000 cells/mm^3^ (data were not available for 14 patients), and 56% had leukocytosis (>11,500 leukocytes/mm^3^) (Technical Appendix Table 3). Diarrheal illness was reported for 83% of HUS patients (bloody diarrhea for 30%); median time from onset of diarrhea to diagnosis of HUS was 6 days (data available for 37 patients). Diarrheal illness in family members was recorded in only 2 HUS cases. One patient had a relapse complicated by bacteremia (*8*), 1 patient died after myocardial complication with pancreatic abscess from which an O80:H2 EHEC strain was isolated, and 1 patient died from septic shock with intestinal necrosis and peritonitis. Eight (17%) cases of neurologic complication (17%) were reported. All the neurologic complications were seizures (documented in 6 cases by imaging) with ischemic strokes. Among the 8 patients with neurologic complications, 2 died, and 1 has difficulty concentrating (19 months after HUS); for 1 patient, the neurologic state could not be assessed, and for 4 others, the neurologic outcome was favorable.

Of the 48 patients HUS, 27% needed acute dialysis support (median duration 10 days, range 3–21 days, IQR 7.4–13.5 days), and 28% had long-term renal sequelae, including proteinuria for all cases, hypertension in 3 cases, and chronic renal failure in 1 case (median follow-up duration 8 months, range 1–108 months, IQR 2–41 months). Finally, only 21% of medical records mentioned the possible source of infection, and no hypothesis could be formulated. 

Genetic characterization showed that all O80:H2 strains of human origin collected in France carried the *stx*2 genes and no *stx*1 genes (Technical Appendix Table 3). The *stx2* subtype could not be determined for the 2 strains (isolated in 2006 and in 2010) because they had lost their *stx2* gene despite preservation at −80°C. Among the remaining strains, 69% had a combination of *stx2* variants, *stx2c/2d* (62%) and *stx2a/2d* (7%); 31% harbored unique variants, *stx2a* (22%) and *stx2d* (9%). All strains had the intimin encoding gene *eae* and its variant *eae*-ξ, and 87% carried the enterohemolysin *ehxA* gene. All 57 strains shared >4 characteristic genes of the pS88 plasmid, *sitA*,* cia*,* hlyF*, and *ompT_p_*; 98% had the *iss* and *iroN* genes; 96% had the *cvaA* gene; and 61% had the *iucC* and *etsC* genes (Technical Appendix Table 3).

Antimicrobial drug susceptibility testing revealed that most strains were multidrug resistant; rates of resistance were 91% for amoxicillin, 89% for nalidixic acid, 82% for cotrimoxazole, and 71% for kanamycin (Technical Appendix Table 3). Overall, 52% of the strains were resistant to all 4 antibiotics.

To examine whether an animal could be the potential source of EHEC O80:H2, we solicited the Laboratoire National de Réference pour les *Escherichia coli*. Only 1 strain from an animal source (LNR-511-4, isolated from raw cow milk cheese) was available. This strain carried *eae-*ξ, *ehxA*, and *stx*2a genes and 7 genes associated with the pS88 plasmid related to ExPEC (*sitA, cia, iss, iroN, hlyF, cvaA*, and *ompT_p_*). This strain also was resistant to amoxicillin, kanamycin, and nalidixic acid.

To investigate the distribution in Europe of this emerging EHEC serogroup, several national reference laboratories in Europe were associated with this study and were asked to send us their available EHEC O80 strains. We obtained 5 strains from Spain, whereas Italy and Germany had no such strain in their collections. Among the 5 strains from Spain, 3 were of human origin (IH42632/03a, IH33264/ 07a, and IH102878/12a) and 2 were of animal origin (VTB-262 from a cow and FV4476 from a pig [the pig-origin strain was actually isolated in Slovakia]) (*28*). All 5 strains shared *eae*-ξ, but *stx2* (*stx*2a) was found in only 2 strains (both of human origin), and none had *ehx*A. Only the 3 human strains harbored most of the investigated genes associated with pS88. The contrast between animal strains and human strains from the national reference laboratory in Spain was also evident in their antibiotic-resistance profiles; only the human strains were multidrug resistant (Technical Appendix Table 3).

**Figure 3:**
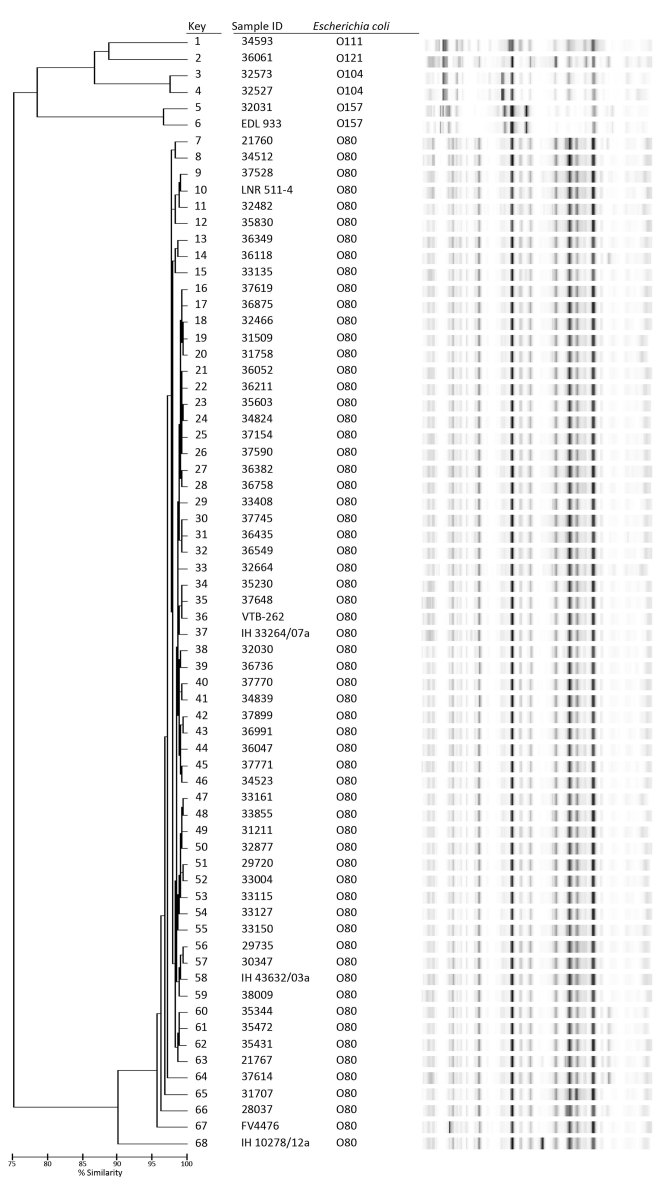
Dendrogram obtained after DiversiLab genotyping analysis (based on PCR amplification of repeat sequences of DNA) of 56 enterohemorrhagic *Escherichia coli* (EHEC) O80 strains from humans in France compared with other isolates detected in France, Germany, and Spain, January 2005–October 2014. Other isolates include 1 animal-origin strain from France (LNR511-4, bovine, 2012); 5 animal- and human-origin isolates from Spain (FV4476, porcine; VTB-262, bovine; IH43632/03a, IH33264/07a, and IH 102878/12a, human); and 6 comparison strains from other serogroups (EDL933 and 32031, O157; 32527 and 32573, O104, isolated during a 2011 outbreak in Germany; 36061, O121; and 34593, O111). ID, identification.

To analyze the genetic diversity and genetic relatedness with other EHEC serogroups, all the O80:H2 strains were analyzed by using rep-PCR and compared with some other serogroups (O157, O104, O111, and O121) (Figure 3). All EHEC O80:H2 strains shared 95% similarity except strain IH102878/12a from Spain (90% similarity). These data suggest that almost all EHEC O80 strains belong to a unique clonal group, regardless of their geographic origin and source (human or animal). EHEC O80:H2 strains were genetically distant to representative strains of serogroups O157, O104, O111, and O121.

**Figure 4:**
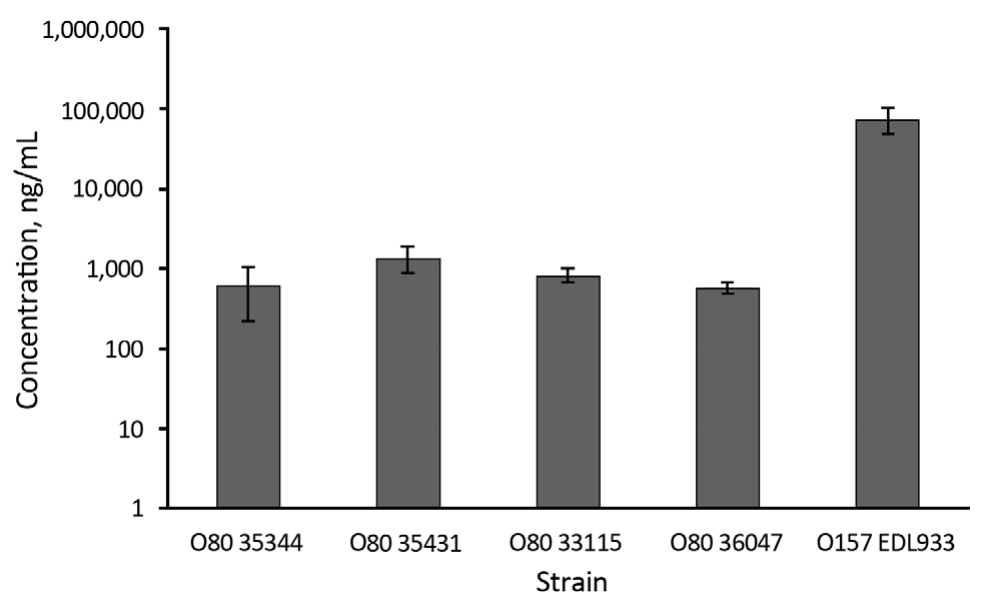
Mean concentrations (logarithmic scale) of Shiga toxin produced in the absence of antibiotics by selected strains of enterohemorrhagic *Escherichia coli* serotypes O80, France, January 2005–October 2014. O157 reference strain (EDL933) was used as control. Error bars indicate SDs.

To study the effect of antibiotics on Stx production in O80:H2 EHEC strains, we selected 4 representative strains (33115, 35344, 35431, and 36047) based on their genotypic and clinical characteristics (Technical AppendixTable 3). Strain EDL933 (O157:H7) served concomitantly as the control. We examined susceptibility to the 3 antibiotics (Table). First, we estimated the basal production of Stx in different strains after 18 h of growth without antibiotics (Figure 4). The basal production among the different O80:H2 strains were comparable but significantly lower compared with that of strain EDL933, which produces ≈100-fold more Stx than certain O80:H2 strains (35344 and 36047). We could not separately estimate the respective rates of Stx1 and Stx2.

**Table:** Antibiotic susceptibility of enterohemorrhagic *Escherichia coli* O80:H2 and O157:H7 isolates used in an antibiotics assay, France, January 2005–October 2014

Isolate (source)	Serotype	MIC, µg/mL		
		Azithromycin	Ciprofloxacin	Ceftriaxone
35344 (stool)	O80:H2	1	0.5	0.25
35431 (blood)	O80:H2	16	0.5	0.06
33115 (pancreas)	O80:H2	16	0.25	0.06
36047 (stool)	O80:H2	16	0.06	0.06
EDL933 (ground beef)	O157:H7	32	0.12	0.12

**Figure 5:**
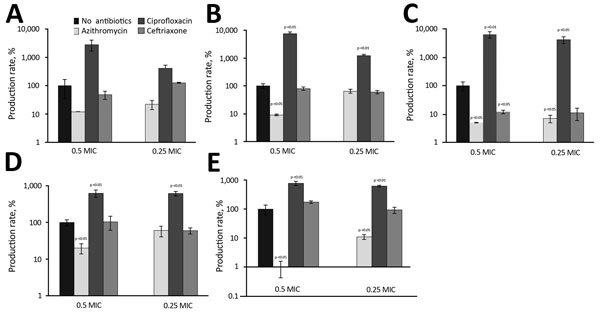
Relative production rate of Shiga toxin produced in 5 strains of enterohemorrhagic *Escherichia coli* (4 O80 strains and 1 O157 strain) at subinhibitory concentrations of azithromycin, ciprofloxacin, and ceftriaxone, compared with basal production rate (no antibiotics), France, January 2005–October 2014. A) Isolate 35344. B) Isolate 33115. C) Isolate 35431. D) Isolate 36047. E) Isolate EDL933. Error bars indicate SDs.

For each strain, we examined the influence of the 3 antibiotics at concentrations below the MICs expressed as relative secretion of Stx compared with basal secretion (without antibiotics) after 18 hours’ incubation period (Figure 5, panels A–E). Overall, azithromycin was responsible for >5-fold decreases of Stx production in all O80:H2 strains at a MIC of 0.5. As expected, the same effect was observed with the EDL933 strain. At all concentrations tested, ciprofloxacin significantly induced a major increase of Stx secretion for all strains except strain 35344. The increase was particularly marked for O80:H2 strains compared with O157 strains; a 100-fold increase was observed for 33115 and 35431 (Figure 5, panels B and C), compared with a 6-fold increase for EDL933 (Figure 5, panel E). Ceftriaxone, in contrast with other antibiotics, did not significantly alter Stx production except for 1 O80:H2 strain (Figure 5, panel C).

**Figure 6:**
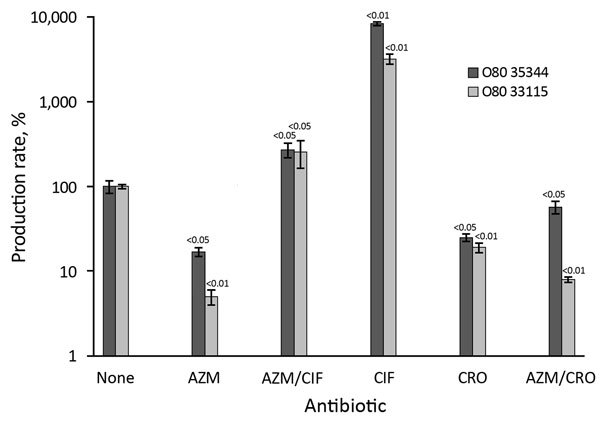
Relative production rate of Shiga toxin produced in 2 strains of enterohemorrhagic *Escherichia coli* O80 (isolates 35344 and 33115) at subinhibitory concentrations of azithromycin, ciprofloxacin, ceftriaxone (alone and in combination), compared to basal production rate (no antibiotics), France, January 2005–October 2014. AZM, azithromycin; AZM/CIF, azithromycin/ciprofloxacin; AZM/CRO, azithromycin/ceftriaxone; CIF, ciprofloxacin; CRO, ceftriaxone. Error bars indicate SDs.

Finally, we wanted to determine whether the beneficial effect of azithromycin on Stx production would persist in the presence of 2 other antibiotics, a combination which might be used in cases of bacteremia. The combinations of antibiotics were tested on 2 O80:H2 strains (Figure 6). Azithromycin paired with ciprofloxacin significantly reduces Stx production compared with ciprofloxacin alone. However, this production was higher than that observed with azithromycin alone, and for both strains, Stx levels were higher for azithromycin/ciprofloxacin compared with no antibiotic. These data indicate that the macrolide might only partially inhibit the noxious effect of ciprofloxacin. The effect of azithromycin in combination with ceftriaxone depended on the strain tested. For strain 35344, the association slightly increased Stx production compared with ceftriaxone, whereas for strain 33115, the opposite was observed. However, in this experiment, the observed Stx rate was lower in ceftriaxone and azithromycin/ceftriaxone assays compared with basal secretion.

## Discussion

In this study, we described the emergence in France of a new virulent EHEC serotype, O80:H2, that harbors singular genetic characteristics of a hybrid STEC/ExPEC pathotype. Several important conclusions can be drawn.

First, the EHEC O80:H2 strain appears to be at least as virulent as the EHEC O157:H7 strains present in France. Indeed, the rate of HUS-associated complications, such as renal injuries (28%) and death (4%), of the O80:H2 strain were comparable with that of O157:H7 (*1*,*4*). Moreover, EHEC O80:H2 isolates have the particular properties to induce invasive infections; >2 of the 53 patients affected had bacteremia or deep abscess. Extraintestinal infections very rarely are associated with EHEC; to our knowledge, only 6 cases of bacteremia have been previously described in the context of HUS (*8*). None of these 6 reports extensively searched for extraintestinal virulence factors. Whether the extraintestinal virulence of EHEC O80:H2 is related to the presence of genetic traits characteristic of an extraintestinal virulence–associated plasmid remains to be determined. Identification of the salmochelin-encoding genes (*iroN*) in 98% of the EHEC O80:H2 from France is of particular interest. This gene is clearly involved in the pathophysiology of *E. coli* bacteremia and meningitis (*9*,*29*).

Because the surveillance system for STEC is voluntary in France and because *stx*-specific PCR is performed only in cases involving diarrhea with HUS suspicion, accurate data are not available on the rates of diarrhea without HUS or of bloodstream infections caused by this serotype and others. Only cases of diarrhea occurring among HUS patient contacts are systematically investigated. Therefore, many cases of O80:H2-related diarrhea are probably undiagnosed, and we cannot draw any conclusion concerning the risk for HUS in cases of diarrhea associated with STEC O80:H2. Moreover, non-HUS cases of bacteremia caused by O80:H2 strains will escape detection.

We were not able to identify the potential source of this emerging EHEC pathotype. All reported cases have been sporadic, and the possibility of foodborne infection remains speculative, even though O80:H2 strains were isolated from few animals. The highest incidence of O80:H2 infection was observed in regions of France where EHEC O157 infections are not predominant, suggesting a possible atypical route or source of infection. Although Spain does not share borders with the high-incidence regions of France, it was the only country where a significant number of EHEC O80:H2 strains were found. Two O80:H2 EHEC strains from Spain (IH102878/12a and IH33264/07a) were very similar to French strains belonging to the same clonal group with a similar virulence genotype, suggesting a direct lineage between the isolates in Spain and France. As is usually observed for other serotypes, most of the infections with these strains occurred during summer and the beginning of autumn (*4*,*7*).

The molecular characterization of the EHEC O80:H2 strains was of particular interest. The presence in all strains from Spain and France of the very rare *eae*-ξ gene combined with results of the genetic diversity study using rep-PCR strongly indicate that these strains, whatever their origin, have a common ancestor combining the O80:H2 serotype and an *eae-*ξ gene containing LEE. On this unique genetic background, several genetic events occurred: the acquisition of >1 prophage encoding Stx, the presence of a plasmid similar to that found in ExPEC, and the presence of a plasmid encoding enterohemolysin. The chronology of these events remains speculative. However, acquisition of Stx2-encoding genes seems to remain particularly active because 3 different variants in >4 different modes (in combination or alone) were observed. The diversity of the extraintestinal virulence plasmidic genes combination suggests the interplay of certain plasmidic determinants and intestinal pathogenic traits. The minimal combination of plasmidic genes common to all strains was the association of *ompT_p_* and *hlyF*, which might represent a beneficial influence on the intestinal pathogenic virulence of *E. coli* O80:H2*.* Chromosomal *ompT* and *hlyF* are involved in the secretion of outer membrane vesicles, which might serve as transporters of toxins and thus might boost the virulence of Stx-producing *E. coli* (*30*,*31*). Whether *ompT_p_* has a similar role remains to be further investigated.

Finally, clinical features and molecular characterization indicating the potential invasive pathogenicity of EHEC O80:H2 raise the question of which antibiotic should be used in such infections. Several clinical studies have suggested a deleterious effect of antibiotics during EHEC infection, leading to recommendations to not use such treatment (*2*). The major explanation of such an adverse effect is the induction of Stx secretion through the SOS response, which is stimulated by antibiotics such as fluoroquinolones in vitro and in experimental models (*32*,*33*). Several studies demonstrated that the effect of antibiotics on HUS depends on their class (*27*,*33*–*37*). Ciprofloxacin raises the production and release of Stx in vitro and is associated with a higher mortality rate in pigs (*33*). Other studies have shown that some antibiotics such as azithromycin might be associated with a decrease of the production and release of Stx in vitro (*37*) and with favorable outcomes in in vivo studies in piglets and mice (*33*,*38*). Finally, during a 2011 outbreak of EHEC O104 infection in Germany, a patient treated by ciprofloxacin plus imipenem unexpectedly had a better prognosis than all others (*14*). This result suggests that response to antibiotics might also differ depending of the strain involved (*34*). 

Our analysis of Stx production in the presence of antibiotics has clearly indicated that ciprofloxacin should not be used in cases of EHEC O80 infection. In contrast, azithromycin provided a beneficial in vitro effect and might be useful in cases of EHEC O80–associated diarrhea. These results are consistent with the systematic review by Agger et al. (*16*), who concluded that a protein synthesis inhibitor can be considered during EHEC infections when specific criteria are met. To our knowledge, only a few studies have tested the effect of antibiotics in combination (*14*,*39*). Our combination assay results would suggest that azithromycin plus ceftriaxone might be a reasonable choice in cases of systemic infection.

These findings should be regarded as preliminary and require confirmation. However, despite these promising in vitro results and because our assays were performed without measuring cytotoxicity, we cannot yet advocate the use of these antibiotics for treatment of patients infected with EHEC O80:H2. However, a planned national clinical trial in France (NCT02336516) to test the efficacy of azithromycin in children with postdiarrheal HUS might soon provide some answers.

In conclusion, a clonal group of EHEC O80:H2 strains of unknown origin and with the ability to induce invasive infections and lethality is emerging in France and represents a new therapeutic challenge. The interplay between intestinal and extraintestinal virulence factors in this new hybrid STEC/ExPEC pathotype remains to be elucidated. Azithromycin might be a possible option to prevent invasive infections caused by EHEC O80:H2, whereas azithromycin/ceftriaxone might be useful in treating such infections.

Technical Appendix Primers used and process applied for the enterohemorrhagic *Escherichia coli *serogroup O multiplex PCR, clinical features of enterohemorrhagic *E. coli* O80:H2 infections in France, and phenotypic and genetic characterization of O80:H2 isolates from France and Spain, January 2005–October 2014.
